# Memory Reinforcement and Attenuation by Activating the Human Locus Coeruleus via Transcutaneous Vagus Nerve Stimulation

**DOI:** 10.3389/fnins.2018.00955

**Published:** 2019-01-09

**Authors:** Niels Hansen

**Affiliations:** Department of Neurodegenerative Diseases and Geriatric Psychiatry, Neurology, University of Bonn Medical Center, Bonn, Germany

**Keywords:** auricular transcutaneous vagus nerve stimulation, memory, locus coeruleus, noradrenaline, hippocampus

## Introduction

Vagus nerve stimulation (VNS) constitutes a standard therapy for treating drug-resistant focal epilepsy (Panebianco et al., 2015) and depression (Aaronson et al., 2013). However, its effects on memory functions and disorders have scarcely been investigated. Three different forms of VNS can be distinguished: invasive vagus nerve stimulation (iVNS) requiring surgery and two non-invasive application forms: (1) auricular transcutaneous vagus stimulation (atVNS) (Frangos et al., 2015) and (2) cervical transcutaneous vagus nerve stimulation (ctVNS) (Zobel et al., 2005). Being non-invasive, economical, and patient-friendly atVNS and ctVNS seem to be superior to iVNS for application in clinics. However, the efficacy between various stimulation types in healthy subjects or patients has not been investigated. In particular, atVNS seems to be easier to use and has been more often investigated than ctVNS. This article therefore focuses on atVNS effects on memory functions in health. In addition, future potential applications in human brain diseases involving memory dysfunctions are delineated.

## Materials and Methods

PubMed was systematically screened for this opinion article in July 2018 applying the items (1) “transcutaneous vagus nerve stimulation” (*n* = 191), (2) “auricular vagus nerve stimulation” (*n* = 123), (3) “non-invasive vagus nerve stimulation” (*n* = 111), (4) “transcutaneous vagus nerve stimulation, and memory” (*n* = 7) (5) “transcutaneous vagus nerve stimulation and locus coeruleus” (*n* = 11), “vagus nerve stimulation and locus coeruleus” (*n* = 70) and “vagus nerve stimulation and memory” (*n* = 90). Relevant articles on this topic were selected based on articles' abstracts (*n* = 15).

## Locus Coeruleus Activation via Transcutaneous Vagus Nerve Stimulation

**Figure 1:**
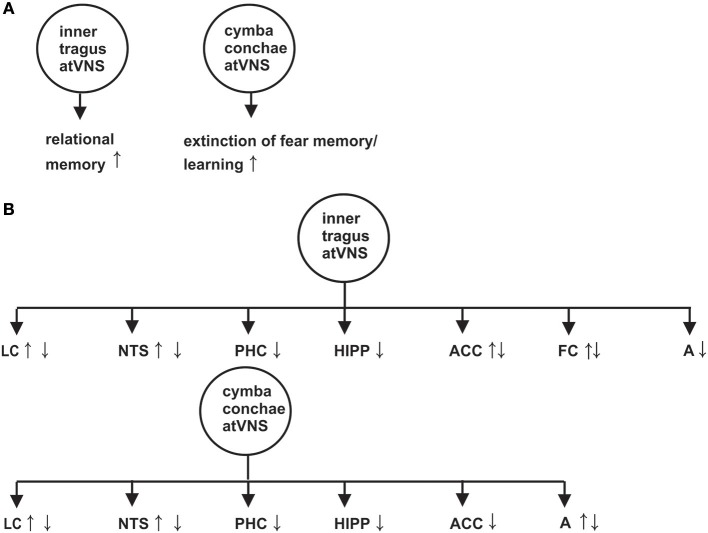
Effect of auricular transcutaneous vagus nerve stimulation on memory functions investigated by distinct memory paradigms in **(A)** and on brain activity revealed by functional magnetic resonance imaging in **(B)**. An arrow with upward direction indicates a greater neuronal activation or reduced neuronal deactivation of the indexed brain structure, whereas an arrow with downward direction suggests a reduced activation or increased neuronal deactivation of the corresponding brain structure. A, amygdala; FC, frontal cortex; Hipp, Hippocampus; LC, Locus coeruleus; NTS, nucleus tractus solitaries; PHC, parahippocampus. The effects on memory and brain activity in this figure are based on the following references (Kraus et al., 2007, 2013; Frangos et al., 2015; Jacobs et al., 2015; Burger et al., 2016, 2017; Yakunina et al., 2017; Badran et al., 2018).

The nucleus tractus solitarius (NTS) is innervated by the vagus nerve and connected to the locus coeruleus (LC) by the nucleus paragigantocellularis (Astier et al., 1990). The NTS-LC can be activated by atVNS (Frangos et al., 2015; Yakunina et al., 2017) or deactivated (Kraus et al., 2013) in humans depending on the atVNS paradigm (Figure 1). The atVNS electrodes can be put either on the inner tragus or the cymba conchae to activate primarily vagus nerve's auricular branch (ABVN) and secondly NTS-LC system, as revealed by functional magnetic resonance imaging (fMRI) (Figure 1) (Frangos et al., 2015; Yakunina et al., 2017). Markers of noradrenergic activity are known to be elevated after moderate atVNS (25 Hz, 1.3 mA) in humans: (1) salivary alpha and (2) memory processing-relevant P300b amplitude of the event-related brain potential (Ventura-Bort et al., 2018) support LC activation via atVNS.

### Activation of Memory Relevant Brain Structures by Transcutaneous Vagus Nerve Stimulation

Anterior inner tragus-atVNS may lead to greater neuronal activation and/or reduced neuronal deactivation of the anterior cingulated cortex and the left frontal cortex (Kraus et al., 2007, 2013; Yakunina et al., 2017; Badran et al., 2018), as revealed in a rised blood-oxygen-level dependent (BOLD) signal within these structures (see Figure 1 for effects of inner tragus-atVNS and cymba conchae-atVNS on memory relevant brain structures). Surprisingly, cymba conchae- and inner tragus-atVNS increases the neuronal deactivation and/or reduces the activation in declarative memory-relevant structures such as hippocampus, parahippocampus, anterior cingulated cortex, frontal cortex and amygdala, as is evident in the weaker BOLD signal in these structures (Kraus et al., 2007, 2013; Frangos et al., 2015; Yakunina et al., 2017). Functionally speaking, the net decrease in neuronal responses in these structures might reflect lateral inhibition to prioritize neuronal activity in a subregion relevant for memory processing (e.g., the dentate gyrus).

### Advance in Transcutaneous Vagus Nerve Stimulation Protocols

Cymba conchae-atVNS seems to be the more suitable position for activating the LC-NTS rather than the inner tragus or inferiorposterior wall of the meatus externus (Yakunina et al., 2017). Their findings are in accordance with study results proving that the cymba conchae is the auricular area supplied completely with the ABVN, whereas the tragus receives less than half thereof (Peuker and Filler, 2002). Although the ABVN innervates the inner tragus only moderately, vagal afferents are activated by atVNS, as is evident in fMRI (Badran et al., 2018), thus other atVNS parameters apart from stimulus location might be decisive for its effect. Rodent experiments showed that a stronger firing rate of LC neurons is caused by a greater stimulus intensity (range: 0.1–2.5 mA) combined with a long pulse width (Hulsey et al., 2017), whereas another study proved an optimal stimulus strength for the memory-augmenting effect between a stimulus intensity of 0.4–0.8 mA (Borland et al., 2016). To apply these findings to humans, a moderate instead of a strong stimulus intensity and long pulse width should be used for optimal atVNS to modulate neuronal brain activity in memory processing structures.

### Memory Modulation via Trancutaneous Vagus Nerve Stimulation by Activating the Locus Coeruleus

The hippocampus features a crucial function in memory formation for events and facts (declarative memory) (Tulving and Markowitsch, 1998; Deuker et al., 2016). VNS in rats is known to potentiate long-term potentiation (LTP) in dentate gyrus synapses (Zuo et al., 2007), a pivotal relay station for information processing within the hippocampus. LTP is a potential neuronal correlate of memory formation (Teyler and Discenna, 1984). In light of these factors, VNS probably modulates neuronal correlates of memory storage. VNS led to increased noradrenaline in the hippocampus in rodents (Raedt et al., 2011), suggesting that VNS activated the LC as a major origin of noradrenaline (NA) in the brain (Amaral and Sinnamon, 1977). Studies in humans support the LC's important role in arousal-enhanced and -prioritized episodic memory formation (Clewett et al., 2018). Based on evidence from those studies, activation or deactivation of the noradrenergic LC system via atVNS would seem to be a potential method for modulating human episodic memory. The amygdala (Fullana et al., 2018; Orsi et al., 2018) and amygdalohippocampal neuronal ensembles (Phelps, 2004; Chaaya et al., 2018) are critically involved in fear memories. Thus the amygdala's activation via atVNS (Liu et al., 2016) is important for extinguishing fear memory.

### Augmentation of Hippocampus-Dependent Relational Memory

Memory formation of associations between items (relational memory) is processed within the hippocampus (Cohen et al., 1997; Horecka et al., 2018) and is known to be modulated by atVNS in a study utilizing a face-name associative memory paradigm (Jacobs et al., 2015). In that study, atVNS (5 mA, 200 μs) was applied during the encoding and consolidation of face-name associations. More accurate face-name associations (hits) were observed after atVNS than sham atVNS (Jacobs et al., 2015). Thus, atVNS seems to augment relational memory function in humans (Figure 1).

### Facilitation of Learning Fear Extinction and the Attenuation of Fear Learning

Neuronal assemblies between the amygdala, hippocampus, anterior cingulated cortex, and ventromedial prefrontal cortex are important for consolidating and extinguishing fear memory (Fullana et al., 2018; Marek and Sah, 2018). A neuronal correlate of posttraumatic stress disorder (PTSD) is impaired fear-memory extinction. Noradrenaline plays a major role in the pathogenesis of PTSD (Hendrickson and Raskind, 2016). AtVNS via LC activation might strengthen the impaired LC-dependent noradrenergic transmission in PTSD modulating fear-memory extinction. Experimental animal evidence suggests that extinction-memory impairment in rats with PTSD-like behavior is reversible by applying iVNS. In addition, PTSD-like behavior in rats (e.g., hyperarousal) can be attenuated by iVNS (Noble et al., 2017). However, to date, the atVNS effect on extinction memory has only been investigated in healthy subjects. Extinction memory can be facilitated in healthy subjects, as two recent studies showed (Burger et al., 2016, 2017). Similar concha cymbaatVNS parameters were utilized in both studies (25Hz, ≤ 0.5mA) (Burger et al., 2016, 2017), and fear-extinction learning in healthy students was facilitated (Burger et al., 2016) (Figure 1). However, the storage of extinction memory one day later was unaffected by atVNS (Burger et al., 2016). Another working group demonstrated no atVNS-dependent modulation of anxiety extinction (Genheimer et al., 2017) being likely based on various stimulation parameter such as mean intensity (1.2mA) (Genheimer et al., 2017) and timing of atVNS. Overall, these studies reveal promising potential for atVNS as a tool for modulating extinction memory in anxiety disorders.

### Memory Modulation by Invasive Vagus Nerve Stimulation in Brain Diseases Involving Memory Dysfunction

Two main brain diseases often characterized by memory and cognitive dysfunctions are temporal lobe epilepsy (TLE) as well as Alzheimer's disease (AD). There is evidence that iVNS improves memory functions in AD patients, as revealed by higher or not worse ADAS-cog and MMSE scores within the disease's distinct time courses (Sjögren et al., 2002; Merrill et al., 2006). MMSE and ADAS-cog scores reflect several aspects of memory-related dysfunctions (Magni et al., 1996; Alexander et al., 2016). Nonetheless, as there was no sham condition, memory fluctuations might explain some of the patients' test results. More investigations have addressed the impact of iVNS on declarative memory function in conjunction with epilepsy (Helmstaedter et al., 2001; Ghacibeh et al., 2006). A benefit from iVNS was observed in epilepsy patients in several memory aspects, i.e., figural (Helmstaedter et al., 2001), verbal (Clark et al., 1999) and working memory (Sun et al., 2017) as well as subjective memory performance (McGlone et al., 2008).

### Future Challenges and Perspectives for Transcutaneous Vagus Nerve Stimulation

Summing up, a few pilot studies in healthy subjects have demonstrated atVNS' potential for memory augmentation, the facilitation of fear-extinction learning, and the attenuation of fear learning by increasing extinction in healthy adults. However, although no investigation has examined the effects of atVNS in human memory disorders to date, iVNS research has revealed beneficial effects from iVNS on memory function in patients with AD and TLE. In particular, the modulation of extinction memory is an interesting candidate for atVNS, as an optogenetic investigation revealed that fear extinction memory is dependent on a depotentiation—or long-term depression (LTD)-like mechanism (Nabavi et al., 2014). LTD is a potential mechanism of memory formation (Kemp and Manahan-Vaughan, 2007; Dietz and Manahan-Vaughan, 2017). Thus, LC activation via atVNS might induce an LTD-like mechanism in fear-related neuronal networks, providing the stimulation protocol is adequate. Indeed, high-frequent electrical LC stimulation in rats induced an LTD in the dentate gyrus (Hansen and Manahan-Vaughan, 2015). Therefore, stimulation parameters from animal investigations should be considered in future human studies.

## Concluding Remarks

Taken together atVNS is a safe, well-tolerated, inexpensive, and handy tool with which to modulate fear and declarative memory function in adults. Further large-scale, randomized, and sham-controlled investigations are warranted to assess whether atVNS can significantly alleviate memory dysfunction incorporating demand stimulation considering disease states and task performance in brain diseases.

## Author Contributions

The author confirms being the sole contributor of this work and has approved it for publication.

### Conflict of Interest Statement

The author declares that the research was conducted in the absence of any commercial or financial relationships that could be construed as a potential conflict of interest.
