# Corrigendum: Memory Reinforcement and Attenuation by Activating the Human Locus Coeruleus via Transcutaneous Vagus Nerve Stimulation

**DOI:** 10.3389/fnins.2019.00186

**Published:** 2019-03-13

**Authors:** Niels Hansen

**Affiliations:** Department of Neurodegenerative Diseases and Geriatric Psychiatry, Neurology, University of Bonn Medical Center, Bonn, Germany

**Keywords:** auricular transcutaneous vagus nerve stimulation, memory, locus coeruleus, noradrenaline, hippocampus

In the original article, there was an error. The stimulation intensity and the atVNS effects on anxiety extinction, were incorrectly stated.

A correction has been made to the section **Locus Coeruleus Activation via Transcutaneous Vagus Nerve Stimulation**, subsection **Facilitation of Learning Fear Extinction and the Attenuation of Fear Learning**:

“Neuronal assemblies between the amygdala, hippocampus, anterior cingulated cortex, and ventromedial prefrontal cortex are important for consolidating and extinguishing fear memory (Fullana et al., 2018; Marek and Sah, 2018). A neuronal correlate of posttraumatic stress disorder (PTSD) is impaired fear-memory extinction. Noradrenaline plays a major role in the pathogenesis of PTSD (Hendrickson and Raskind, 2016). AtVNS via LC activation might strengthen the impaired LC-dependent noradrenergic transmission in PTSD modulating fear-memory extinction. Experimental animal evidence suggests that extinction-memory impairment in rats with PTSD-like behavior is reversible by applying iVNS. In addition, PTSD-like behavior in rats (e.g., hyperarousal) can be attenuated by iVNS (Noble et al., 2017). However, to date, the atVNS effect on extinction memory has only been investigated in healthy subjects. Extinction memory can be facilitated in healthy subjects, as two recent studies showed (Burger et al., 2016, 2017). Similar concha cymba-atVNS parameters were utilized in both studies (25 Hz, ≤ 0.5 mA) (Burger et al., 2016, 2017), and fear-extinction learning in healthy students was facilitated (Burger et al., 2016) (Figure 1). However, the storage of extinction memory one day later was unaffected by atVNS (Burger et al., 2016). Another working group demonstrated no atVNS-dependent modulation of anxiety extinction (Genheimer et al., 2017) being likely based on various stimulation parameter such as mean intensity (1.2 mA) (Genheimer et al., 2017) and timing of atVNS. Overall, these studies reveal promising potential for atVNS as a tool for modulating extinction memory in anxiety disorders”.

The authors apologize for this error and state that this does not change the scientific conclusions of the article in any way. The original article has been updated.
